# The Redox Process in Red Blood Cells: Balancing Oxidants and Antioxidants

**DOI:** 10.3390/antiox14010036

**Published:** 2024-12-31

**Authors:** Dala N. Daraghmeh, Rafik Karaman

**Affiliations:** Pharmaceutical Sciences Department, Faculty of Pharmacy, Al-Quds University, Jerusalem P.O. Box 20002, Palestine; rkaraman@staff.alquds.edu

**Keywords:** red blood cells, oxidant, antioxidant, oxygen, hemoglobin

## Abstract

Red blood cells (RBCs) are a vital component of the body’s oxygen supply system. In addition to being pro-oxidants, they are also essential components of the body’s antioxidant defense mechanism. RBCs are susceptible to both endogenous and exogenous sources of oxidants. Oxyhemoglobin autoxidation is the primary source of endogenous RBC oxidant production, which produces superoxide radicals and hydrogen peroxide. Potent exogenous oxidants from other blood cells and the surrounding endothelium can also enter RBCs. Both enzymatic (like glutathione peroxidase) and non-enzymatic (like glutathione) mechanisms can neutralize oxidants. These systems are generally referred to as oxidant scavengers or antioxidants, and they work to neutralize these harmful molecules (i.e., oxidants). While their antioxidative capabilities are essential to their physiological functions and delivering oxygen to tissues, their pro-oxidant behavior plays a part in several human pathologies. The redox-related changes in RBCs can have an impact on their function and fate. The balance between pro-oxidants and antioxidants determines the oxidative status of cells, which affects signal transduction, differentiation, and proliferation. When pro-oxidant activity exceeds antioxidative capacity, oxidative stress occurs, leading to cytotoxicity. This type of stress has been linked to various pathologies, including hemolytic anemia. This review compiles the most recent literature investigating the connections between RBC redox biochemistry, antioxidants, and diverse disorders.

## 1. Introduction

Red blood cells (RBCs), also known as erythrocyte, are traditionally considered the most prevalent cells in the body, excluding the microbiome, with an estimated average of 25 trillion cells per average individual [1,2]. The primary function of RBCs is to transport hemoglobin (Hb), which carries oxygen (O_2_) from the lungs to the tissues and carbon dioxide (CO_2_) from the tissues to the lungs [3]. Moreover, these cells significantly contribute to regulating acid-base homeostasis for the whole blood [3]. These functions are interconnected to each other; the binding affinity of O_2_ to the ferrous heme (Fe^2+^) of Hb is subject to regulation by oxygen partial pressure, acid/base equilibria (PH), and the levels of 2,3-diphosphoglycerate. To keep Hb working efficiently, it must remain in a reduced state, as, if the Fe^2+^ within its prosthetic group is oxidized to ferric (Fe^+3^) heme, it forms methemoglobin (metHb), which dramatically decreases the Hb’s oxygen affinity [4].

Maintaining the high intracellular concentration of Hb is crucial in transporting oxygen to the body’s tissues. However, this process comes with a risk: Hb autoxidation generates harmful superoxide radicals [5]. Reactive species are also generated in the vasculature, such as the endothelium, and neutrophils and macrophages can also diffuse and impact RBCs [6]. To combat this, RBCs employ antioxidants. Consequently, RBCs display both antioxidant and pro-oxidant activities; an imbalance between these forces can lead to oxidative stress (OS), which is associated with various health problems, including hemolytic anemia [1,7,8]. Under normal physiological conditions, RBCs typically favor antioxidant activity; but, in different human pathologies, pro-oxidant activity may take precedence [1,7]. It is important to recognize that this dual behavior can be influenced by both internal changes and external factors [1].

RBCs face several challenges in sustaining their functionality. These include elevated levels of oxidants and iron, coupled with their limited ability to repair damaged components. This vulnerability arises because mature RBCs lack nuclei and organelles, such as mitochondria, making them especially prone to OS. To protect RBC function and integrity, robust antioxidant systems are necessary. The antioxidant systems in RBCs, mainly the glutathione and thioredoxin systems, protect against OS using NADPH, which is generated through the oxidative pentose phosphate pathway (PPP). While glutathione (GSH) is not a direct antioxidant, glutathione reductase plays a crucial role in keeping reduced glutathione available for redox balance. The thioredoxin system, comprising NADPH, thioredoxin reductase, and thioredoxin, is responsible for regulating protein balance and eliminating ROS. Furthermore, it acts as a complementary system to glutathione, emphasizing the interconnected nature of these antioxidant defenses [8].

Maintaining the redox status of RBCs is crucial not only to ensure an adequate oxygen supply to every tissue cell, but also to maintain a healthy circulatory system through RBC interactions with other blood cells and the vascular endothelium [1,7]. In this review, we explored the importance of redox regulation in RBCs for preserving cell functionality and integrity. This includes examining the origins of ROS and enzymatic and non-enzymatic antioxidant mechanisms, and the consequences of redox state dysregulation.

## 2. Methodology

PubMed, Embase, and Google were searched for English-language full-text articles using the following terms: red blood cells, oxidant, antioxidant, reactive oxygen species, reactive nitrogen species, hemoglobin, RBC disease, sickle cell disease.

The references included in the manuscript were selected from the publications identified according to the authors’ opinion of their relevance to the topic of this review published between 2019 and 2024.

## 3. Results

### 3.1. Human RBCs

Blood is a mixture of different components, each with a distinct function. RBCs make up 40–50% of the blood, while white blood cells comprise only about 1%, including peripheral blood mononuclear cells and polymorphonuclear cells. The remaining 55% is plasma [9]. RBCs are a critical component of blood, contributing to blood rheology, tissue perfusion efficiency, and gas exchange [5,10].

The cytosol in RBCs is primarily composed of Hb, followed by carbonic anhydrase and Peroxiredoxin-2 [11]. Additionally, other proteins focused on oxidant detoxification and glucose metabolism are present. Notably, RBCs differ from other cells as they do not have organelles such as the nucleus and mitochondria, the primary site of cellular respiration [8]. Therefore, mature erythrocytes cannot synthesize new proteins and rely exclusively on the anaerobic breakdown of glucose through the glycolytic pathway to generate their energy. Glucose is the primary source of ATP, while NADH and NADPH serve as sources of reducing equivalents, as they are derived from glycolysis and the PPP, respectively [12]. It is essential to maintain transmembrane ion gradients, membrane integrity, and cytoskeleton interaction, in which ATP plays a crucial role in. Maintaining the biconcave shape of RBCs is essential, enabling them to circulate through capillaries while preventing hemolysis that could release Hb into the intravascular space, causing harmful effects [11].

### 3.2. Redox Reaction

**Figure 1:**
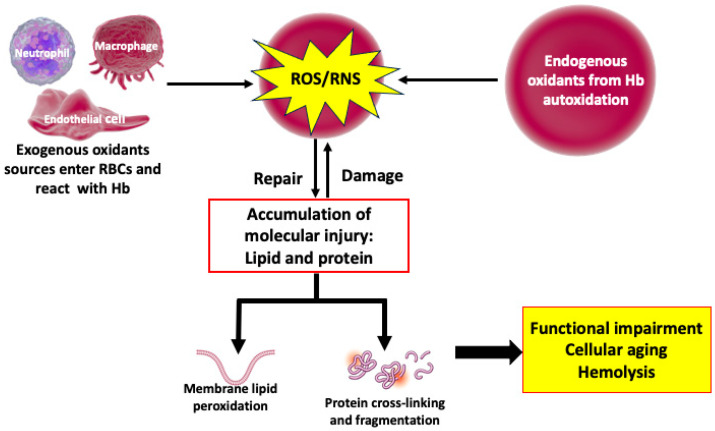
The effects of reactive oxygen/nitrogen species on the body. Endogenous and exogenous sources of ROS/RNS can lead to protein, lipid, and DNA damage and accumulation when the body’s defensive or repair mechanisms weaken. ROS/RNS: Reactive oxygen/nitrogen species.

Redox reactions play a critical role in various cellular processes such as biosynthesis and regulation. They are key to understanding biological oxidation, radical effects, and antioxidants. RBCs have complex redox systems that are crucial for maintaining cellular integrity, regulating cellular metabolism, and influencing cellular shape and flexibility. ROS and RNS play a dual role in RBCs, exhibiting both beneficial and harmful effects. These molecules are essential in normal physiological processes including cellular signaling and maintaining redox balance. However, their overproduction can lead to OS, causing cellular damage and contributing to various pathological conditions (Figure 1) [13].

Thiols, especially cysteine residues in proteins, serve as significant targets for ROS/RNS-mediated signaling because of their capacity for reversible oxidation. The reactivity of different ROS/RNS with thiols varies significantly, with superoxide radical reaction rates for different thiols ranging from 1.2 × 10^5^ to 4.8 × 10^5^ M^−1^s^−1^ [14].

Molecular oxygen undergoes a four-electron addition process to generate water in the mitochondria. However, small amounts of short-lived reactive toxic intermediates are produced when oxygen is partially reduced, including superoxide (O_2_·). Superoxide dismutase (SOD) enzyme helps convert O_2_· to hydrogen peroxide (H_2_O_2_). H_2_O_2_ is more stable than O_2_· and can easily cross biological membranes. When present with metals like Fe^2+^, H_2_O_2_ is converted to into a highly reactive hydroxyl radical ·OH. These free radicals are reactive molecules that occur naturally in the human body during metabolic reactions. Living systems can contain various types of free radicals, including oxygen and nitrogen radicals (i.e., ROS and RNS) (Table 1) [15].

#### 3.2.1. Sources of Reactive Species (Oxidants) in RBCs

The ROS/RNS are not a single chemical oxidant species; rather, they are a diverse group of molecules that are responsible for a wide range of biological functions [8]. While the mitochondrion is the primary source of ROS in most cells [16], mature RBCs lack mitochondria, and, instead, the oxygen carrier protein, Hb, is the major source of ROS, produced as a result of autoxidation. Methemoglobin generation (from auto-oxidation and oxidation) and hemoglobin reduction are balanced to determine the amount of circulating methemoglobin in healthy persons. This literature review emphasizes how intricate the oxidation and reduction mechanisms that guarantee hemoglobin molecules’ ability to carry oxygen are. RBCs’ reduction and antioxidant mechanisms, which interact with intraglobular hemoglobin, maintain a low level of circulating methemoglobin. SOD, glutathione peroxidase, gluthathione, ascorbic acid, and other reducing agents and antioxidants are also present in plasma. They regulate the physiologically low extracellular hemoglobin concentration and have very low concentrations

RBCs are continuously exposed to both internal and external ROS/RNS (Figure 1), which are important in regulating their physiological function and supply of oxygen to the tissues [8]. ROS/RNS also serve as a signaling molecule. However, excess amounts of these compounds can be detrimental to cellular homeostasis due to their highly oxidizing nature [17].

The oxidation tendency is a crucial property of haem proteins. The functional state of the haem group, Fe^II^, is vital for carrier haem proteins such as Hb, which binds to oxygen. However, instability occurs when the ion shifts to the ferric form (Fe^3+^), converting it to metHb. This change reduces its oxygen-binding capacity. Approximately every 24 h, about 3% of total Hb undergoes autoxidation, leading to the gradual production of metHb within cells, limiting oxygen transport [18]. Specific drugs like benzocaine and nitrite can accelerate this process [19]. The superoxide radical generated during this process can further convert it into (O_2_^−^) and hydrogen peroxide (H_2_O_2_). Healthy RBCs promptly convert metHb back into Hb with the help of NADH-cytochrome b5 reductase, supported by reductants like NADPH, glutathione, and ascorbic acid to prevent adverse reactions [8,20]. Together, these components utilize NADH to reduce haem iron [19,20]. Genetic factors can also influence metHb formation [19,20].

One such source of ROS is Hb’s non-enzymatic and enzymatic degradation, which produces biliverdin, carbon monoxide, and iron ions (Fe^2+^). Free iron, if not scavenged by ferritin, can catalyze free radical reactions (i.e., Haber–Weiss (Equations (1) and (2)) and Fenton reactions (Equation (2))) producing hydroxyl radicals (OH^−^). Ferritin is a key player in the removal of free iron within RBCs, which ultimately reduces the likelihood of the Haber–Weiss reaction occurring in the cell [21,22]. Additionally, RBCs can produce NO under both normoxic and hypoxic conditions, which can react with O_2_^·−^ to produce Peroxynitrite [8].

Fe^+3^ + O_2_^−·^ → Fe^+2^ + O_2_ (1)

Fe^+2^ + H_2_O_2_ → Fe^+3^ + ^·^OH + OH^−^ (2)

In blood circulation, leukocytes, primarily neutrophils and macrophages, release ROS into plasma. While a significant number of ROS are released from neutrophils and macrophages, not all are taken up by RBCs. In fact, many of these reactive molecules are neutralized in the plasma before they can even reach the RBCs. However, in the microcirculation, where RBCs are in close proximity to the vasculature and Hb becomes partially oxygenated, ROS released from neutrophils, macrophages, and endothelial cells can be taken up by RBCs. Once they are inside RBCs, they are neutralized by RBC’s antioxidant system. In fact, the addition of hydrogen peroxide to RBCs quickly reacts with catalase, transforming it into oxygen without oxidizing Hb [23].

#### 3.2.2. Reactive Species Derived from RBCs and Other Sources in the Vasculature

##### Superoxide

Superoxide anion (O_2_^−^) is formed when dioxygen (a molecule with two unpaired electrons) accepts one electron to complete an orbital, resulting in one unpaired electron and a net negative charge [24]. Intracellularly, as a result of the transfer of an electron that reduces oxygen in the respiratory chain, superoxide (O_2_^·–^) is produced as the primary species. The reduction in oxygen results in the formation of superoxide, a product of single-electron reduction. In RBCs, oxyHb autoxidation can lead to the formation of both O_2_^·–^ and metHb (Hb (Fe^+3^)).

Hb (Fe^+2^ − O_2_) → Hb (Fe^+3^) + O_2_^−·^ (3)

This O_2_^−^ is short-lived and is either converted spontaneously or enzymatically by SODs to H_2_O_2_, which is more stable. The superoxide radical undergoes a dismutation reaction with another superoxide radical (Equation (4)). This results in the oxidation of one radical to oxygen, and the reduction in the other to hydrogen peroxide [24]. Depending on the pH level, superoxide exists in equilibrium with hydro-peroxyl (HO_2_), which can easily diffuse in lipids. However, under physiological pH conditions, the anion is predominant.

O_2_ + O_2_^·−^ + 2H_2_O − SOD → H_2_O_2_ + O_2_ (4)

##### Hydrogen Peroxide

Hydrogen peroxide (H_2_O_2_) is a non-radical ROS composed of two linked oxygen atoms through a single bond. The production of H_2_O_2_ is predominantly stimulated by plasma membrane NADPH oxidases and by the conversion of superoxide anion by SOD (Equation (4)). Acting as an oxidizing agent, H_2_O_2_ exhibits a relatively high specificity towards specific protein cysteine thiols and interacts with Fe-S clusters. Even at low concentrations of 10 μM, hydrogen peroxide can still inflict damage on cells. However, at higher levels, these substances have the ability to inactivate enzymes responsible for producing cellular energy, such as glyceraldehyde-3-phosphate dehydrogenase [25]. It can cross membranes directly or utilize aquaporin channels and is generally harmful to cells. Catalase, glutathione peroxidase, and peroxiredoxins are the main enzymes known for their ability to remove hydrogen peroxide [26].

##### Hydroxyl Radical

Hydroxyl radical (OH^−^) is a free radical that is highly reactive and exists in a neutral state as the hydroxide ion [27]. It is an incredibly reactive species that can inflict damage on organic and inorganic molecules, such as proteins, lipids, DNA, and carbohydrates. Compared to other ROS produced in cells, it is considered the most hazardous [27]. Unlike other ROS, there are no enzymes that can neutralize OH^−^.

Two processes generate the hydroxyl radical. The first is the Fenton reaction (Equation (2)), where OH^−^ is produced from H_2_O_2_ in the presence of free Fe^2+^. During periods of stress, excessive amounts of oxygen can release free iron from ferritin, triggering the Fenton reaction, which creates OH^·^. The second process is the Haber–Weiss reaction; the iron (Fe^3+^) oxidized in the Fenton reaction is then reduced back to Fe^2+^ through a reaction with O_2_^−^, allowing it to engage in redox cycling again (Equation (2)) [21,22]. With its high reactivity, OH^−^, as a non-specific oxidant, swiftly interacts with nearby targets like proteins and lipids within a nanometer radius at its source. This rapid reaction leads to its short intracellular half-life, lasting approximately 10^−9^ seconds.

##### Nitric Oxide

Nitric oxide (NO^−^) is a radical molecule that is produced intracellularly from L-arginine by nitric oxide synthases (NOS), which are specific enzymes. This reaction requires molecular oxygen and NADPH as reducing equivalents. NO^−^ acts as an autocrine and paracrine signaling molecule with a multitude of benefits, including promoting vascular relaxation, inhibiting platelet aggregation, reducing inflammation, and modulating neural activity. NOSs are complicated enzymes expressed in endothelial cells (NOS3) and leukocytes (NOS2). Recently, an endogenous RBC NOS_3_ has been discovered, which is present in low abundance, and whose function remains poorly understood. Endothelial NOS3 responds to changes in Ca^2+^ concentration to produce NO·, which can diffuse through cell membranes to cause relaxation. NO· also reacts with oxyHb in RBCs to yield nitrate and metHb, and the diffusion across the RBC-free layer is the primary obstacle to NO consumption by RBCs [28,29].

Hb (Fe^+2^ − O_2_) + NO^−^ → Hb (Fe^+3^) + NO_3_^−^ (5)

NO^−^ may react with O_2_^·−^ to form the potent oxidizing agent peroxynitrite (ONOO^−^).

##### Peroxynitrite

The peroxynitrite anion is formed through the reaction between two radicals, O_2_^·−^ and NO^·^ (Equation (1)), which occurs at rates that are controlled by diffusion [30].

NO^·^ + O_2_^·−^ → ONOO^−^ (6)

Peroxynitrite is a potent oxidant with the ability to carry one or two electrons. At physiological pH, peroxynitrite will comprise a mixture of ONOO^−^ and the protonated peroxynitrous acid (ONOOH). ONOOH can decay at a relatively slow pace compared to nitric acid, along with a 30% share of HO· and nitrogen dioxide (NO_2_·).

ONOOH → HNO_3_(70%) + [NO_2_· + HO·] (7)

##### Nitrogen Dioxide

The interaction between peroxynitrite and carbon dioxide leads to the formation of nitrogen dioxide and carbonate radical (CO_3_^·−^). Nitrogen dioxide is a powerful oxidant that can easily traverse cellular membranes. It reacts with intracellular thiols and lipids in the membrane. On the other hand, carbonate radicals are unable to pass through cellular membranes and, thus, preferentially react with proteins [31,32].

**Table 1:** List of ROS/RNS in RBCs.

**Radical**	
Superoxide	O_2_^⋅−^
Hydroxyl radical	OH^−^
Nitric oxide	NO^−^
Nitrogen dioxide	NO_2_·
**Non-Radical**	
Hydrogen peroxide	H_2_O_2_
Peroxynitrite	ONOO^−^

## 4. Reactive Species and Cell Damage

While oxygen is critical for energy production, it can also be toxic and mutagenic, threatening cells. Nonetheless, cells have developed antioxidant defenses to counteract these threats, enabling their survival [15]. Overall, the dual nature of oxygen highlights the delicate balance that exists in biological systems. While oxygen is necessary for life, it can also be harmful if not properly regulated. Through their antioxidant defenses, cells have developed ways to mitigate these risks, allowing them to thrive in an oxygen-rich environment [33].

Hb redox reactions generate ROS and RNS species that can damage RBC proteins and lipids. This can occur through both autoxidation and exogenous reactive species that invade the RBC. Over time, this leads to cumulative damage in RBCs, which cannot synthesize new proteins. Hypoxia causes an imbalance between energy supply and demand, potentially resulting in cell death and organ failure. RBCs play a crucial role in modulating OS during hypoxia. Hypoxia increases ROS and RNS production while reducing antioxidant capacity in RBCs, potentially causing membrane protein cross-linking, damage the cytoskeleton and lipid peri-oxidation. These alterations can affect membrane fluidity and increase ion permeability, potentially leading to hemolysis. To adapt to hypoxia, cells coordinate the downregulation of metabolic demand and supply, preventing ATP utilization and production mismatch. NO is a key signaling molecule in hypoxic response, mediating vasodilation to improve blood flow and oxygen supply while modulating energy metabolism [34].

### 4.1. Antioxidant Systems

RBCs have a very basic structure, lacking mitochondria and nucleus, which results in a limited protein synthesis capacity [35]. Despite this, these cells have a relatively long lifespan of approximately 120 days in the bloodstream [9]. High levels of molecular oxygen and iron (found in the heam group of Hb) in RBCs create the potential for the formation of ROS. Due to their limited protein synthesis capacity, RBCs require robust protection against ROS and strong oxidative damage repair mechanisms [5]. To maintain their antioxidant systems, RBCs rely on both enzymatic (endogenous) and non-enzymatic (supplemental/nutritional) mechanisms. Enzymatic mechanisms include catalase (Cat), glutathione peroxidase (GPX), and superoxide dismutase (SOD), while non-enzymatic antioxidants include glutathione and ascorbic acid. These antioxidants, along with molecules that work as redox couples and/or enzyme cofactors such as the critical glutathione, and antioxidant enzymes like glutathione peroxidase, catalase, and superoxide dismutase, are responsible for scavenging and removing ROS from the body [36,37,38].

The enzymatic and non-enzymatic redox antioxidant systems present in the RBCs have a crucial role in maintaining Hb in a reduced state. This is critical for its ability to bind with oxygen. These systems work by controlling the production of metHb, thereby reducing ROS generation. Moreover, they safeguard cellular membrane lipids, proteins, channels, and metabolic enzymes from damage.

#### 4.1.1. Antioxidant Molecules and Redox Couples

Protein S-glutathionylation is a reversible post-translational modification that plays a crucial role in regulating protein function and cellular processes. This modification involves the addition of glutathione to cysteine residues, which can modulate enzyme activity and protein–protein interactions and protect against irreversible oxidation. S-glutathionylation is promoted by oxidative or nitrosative stress and can be regulated by enzymes such as glutathione-S-transferase-P and glutaredoxin. It affects various cellular functions, including cell signaling, metabolism, and apoptosis. The glutathionylation–deglutathionylation cycle interacts with other post-translational modifications, such as phosphorylation, acetylation, and glycosylation, to fine-tune protein function. This modification has been observed in diverse organisms from bacteria to humans, and plays a role in redox signaling in plants. Understanding S-glutathionylation is crucial for elucidating cellular responses to redox changes and stress conditions.

The first category includes antioxidant molecules and redox pairs, such as reduced/oxidized glutathione (GSH), ascorbate/dehydroascorbate, and α-tocopherol [36]. GSH, a linear tripeptide composed of L-glutamine, L-cysteine, and L-glycine, is synthesized by c-glutamine-cysteine ligase and glutathione synthase. Glutathione exists in either the reduced form (GSH) or the oxidized form (GSSG, a dimer of two GSH molecules). In healthy human RBCs, the majority of glutathione exists in the reduced form, GSH, which is vital in reducing ascorbate, oxidized proteins, and oxidized lipids. Enzymes like glutaredoxin (GRx) and GPx utilize GSH as a reducing agent. The GSH/GSSG ratio in RBCs is used to measure the redox state of an organism, with reduced GSH typically constituting 90–95% of the total GSH. Enzymes that use GSH as a reducing equivalent include GRx and GPx [39]. Glutathione reductase is the enzyme responsible for GSH recycling, which converts glutathione disulfide (GSSG) back into the reduced GSH by consuming NADPH [40].

Ascorbate, commonly known as vitamin C and a key reducing agent in RBCs, is either synthesized or resynthesized from dehydroascorbic acid using glutathione in a process catalyzed by various enzymes, particularly the cytoplasmic GRxs. These enzymes not only facilitate the conversion of dehydroascorbic acid, but also play a role in deglutathionylation, protein disulfide reduction, and Fe-S linkage formation. The primary method of transporting ascorbate into RBCs is through the glucose transporter 1 [41]. To preserve redox homeostasis, the redox pair ascorbate/dehydroascorbate diminishes metHb and oxidants that enter into the cell membrane. The plasma membrane redox system (PMRS) is maintained by ascorbate, which facilitates various antioxidant mechanisms by reducing oxidants and metHb. The PMRS transfers electrons from the intracellular cytosolic RBC compartment to the extracellular medium by oxidizing intracellular electron donors like ascorbate and NADH, acting as a compensatory mechanism against elevated ROS/OS, and may contribute to redox regulation throughout the body [42].

The exact physiological significance of PMRS is not fully understood, but studies suggest that increased PMRS activity in aging erythrocytes might be a protective response to enhance extracellular dehydroascorbate reduction and ascorbate recycling under conditions of elevated OS. Consequently, PMRS in RBCs may function as a compensatory mechanism against heightened ROS and OS, playing a role in redox regulation throughout the body [42].

Vitamin E, or α-tocopherol, is another crucial antioxidant found within RBCs’ membranes due to its lipophilic properties. α-Tocopherol plays a critical role in preventing lipid peroxidation due to its strong oxidant-scavenging properties within RBCs. The lipophilic nature of α-tocopherol allows it to accumulate in RBC membranes, limiting the amplification of peroxidation chain reactions [43].

#### 4.1.2. Sources of Redox Equivalents (NADH, NADPH)

Glucose metabolism is crucial for RBC functions through three primary mechanisms. First, RBCs rely solely on glycolysis to produce ATP. Second, around 25% of glucose in RBCs is used to produce the specific metabolite 2,3-bisphosphoglycerate to regulate Hb’s oxygen affinity. Third, RBCs utilize the oxidative branch of the PPP to generate reducing equivalents (NADPH) to maintain glutathione balance and combat OS.

Due to the absence of mitochondria in RBCs, their glucose metabolism is predominantly controlled by glycolysis and the PPP. Glycolysis produces ATP to maintain ion gradients across the membrane, NADH to convert metHb to oxyHb, and pyruvate to lactate. Meanwhile, the PPP produces NADPH, a vital biological carrier of reducing equivalents that commonly acts as a coenzyme. NADPH is prevalent in various cells, where it plays a crucial role in preserving redox balance. In human RBCs, NADPH concentrations typically range from 16 to 44.9 μM.

Redox equivalents, including NADH and NADPH, make up the second category of the antioxidant systems. These equivalents provide reducing counterparts to enzymes that catalyze redox reactions in RBCs. During glycolysis and the PPP, they undergo reduction. The PPP is responsible for converting glucose-6-phosphate (G6P) to NADPH, which is vital in repairing oxidized proteins and protecting against ROS in RBCs [44,45].

Oxygen levels influence RBC metabolism via band 3 protein–anion exchanger 1 (B3p-AE1). The interactions between B3p-AE1 and Hb, along with glycolytic processes, are essential. As the most abundant membrane protein in RBCs, B3p-AE1 is vital for anion exchange and maintaining cellular homeostasis, and it is also a primary target for oxidants. At high oxygen states, B3p inhibits glycolytic enzymes, promoting the PPP and enhancing the generation of reducing equivalent NADPH, which supports related antioxidant systems. Conversely, at low oxygen states, deoxyhemoglobin binds to B3p, releasing glycolytic enzymes from the membrane and increasing glycolysis (enhances energy production in the form of ATP and NADH). Oxygen status can directly influence B3p function, possibly through the oxidation of the transporter or its associated proteins. Maintaining a balance between glycolysis and the PPP is crucial for RBCs to effectively meet their metabolic requirements, including membrane pump function, cytoskeletal integrity, and lipid homeostasis, all of which depend on the oxygen levels present in the lungs or peripheral capillaries [46,47].

It is fascinating how the antioxidant defense system is closely linked to the energy status of RBCs. Prolonged fasting can reduce the levels of reduced NADPH in RBCs due to limited glucose availability for the glycolytic pathway. Furthermore, the absence of riboflavins, essential for the coenzyme flavin adenine dinucleotide (FAD) and crucial for glutathione reductase function, can compromise the antioxidant defense during fasting. NADPH plays a vital role in maintaining the redox balance within RBCs. Any malfunction in enzymes within the PPP can significantly impact overall membrane stability and permeability.

#### 4.1.3. Enzymatic Antioxidant Systems

The third category of RBCs’ antioxidant systems involves enzymatic antioxidant systems that play a vital role in maintaining their functionality. Enzymes that are involved in oxidant processing in mature RBCs are SOD1, Cat, GPx, and Prx2. The SOD enzyme family consists of three isoforms with varying prosthetic group structures (manganese or copper and zinc), compartmentalization, and functional importance. Particularly crucial in mature RBCs is the SOD1 isoform containing copper/zinc, which facilitates the dismutation of the superoxide anions into hydrogen peroxide (Equation (4)). This anion is mainly formed in RBCs by Hb autoxidation (Equation (3)).

Catalases are enzymes that play a crucial role in breaking down hydrogen peroxide into water and oxygen (Equation (8)). There are four main types of catalases, including monofunctional catalases (typical catalases), bifunctional catalase-peroxidases, nonheme catalases, and miscellaneous proteins with minor catalytic activities. Human RBC catalase belongs to the group of monofunctional catalases [48].

2H_2_O_2_ − Cat → −CAT2H_2_O + O_2_ (8)

GPx is a critical component of the thiol-dependent antioxidant systems found in RBCs [35]. In mammals, there are eight different types of glutathione peroxidases (GPx1–GPx8), with GPx1 being the predominant isoform in RBCs. GPx use glutathione (GSH) as a reductant, which helps catalyze H_2_O_2_ into water [37,38].

Thiol-containing substances are essential for protecting the body against oxidative injury and regulating redox signaling. The thiol-dependent antioxidant system comprises glutathione (GSH), thioredoxins (Trxs), glutaredoxins (Grxs), and peroxiredoxins (Prxs) systems, which act as electron donors to create a powerful antioxidant defense system against potential oxidant threat [44,45].

Prxs refer to a collection of sulfhydryl-dependent peroxidases. Among the six known mammalian isozymes of Prxs (Prx1–6), only three are found in RBCs: Prx1, Prx2, and Prx6. Of the three, Prx2 is the most abundant. Prxs are responsible for reducing organic hydroperoxides and H_2_O_2_ to water and alcohols [49,50].

In summary, hydrogen peroxide is converted into oxygen and water by Cat, GPx, or Prx2 enzymes. Typically, under regular circumstances, GPx is the main enzyme responsible for breaking down most of the H_2_O_2_ by transforming reduced GSH into GSSG. GSSG is then restored by GSH reductase using NAPDH. However, in situations of high O_2_^-^ and increased H_2_O_2_ levels, Cat intervenes to convert H_2_O_2_ into water.

### 4.2. Non-Enzymatic Antioxidant System

Non-enzymatic antioxidants can be either produced within the body or obtained from natural sources. They are classified into two categories: water-soluble antioxidants, including vitamin C, lipoic acid, glutathione, and uric acid, and lipid-soluble antioxidants, such as carotene, melatonin, vitamin E, and Coenzyme Q [51,52]. Vitamins C and E play essential roles in protecting against oxidative damage. While vitamin C functions in aqueous environments, vitamin E acts as a chain-breaking antioxidant in lipid phases. Vitamin C is effective in diminishing superoxide and lipid peroxyl radicals while also working synergistically with vitamin E. Uric acid serves as an endogenous antioxidant with metal-chelating properties, scavenging nitrogen radicals and superoxide in plasma, which aids in preventing the formation of peroxynitrite. In RBCs, uric acid neutralizes free radicals and ROS, helping to maintain a smooth membrane surface and inhibit the development of echinocytes [53].

## 5. Oxidative Stress

**Figure 2:**
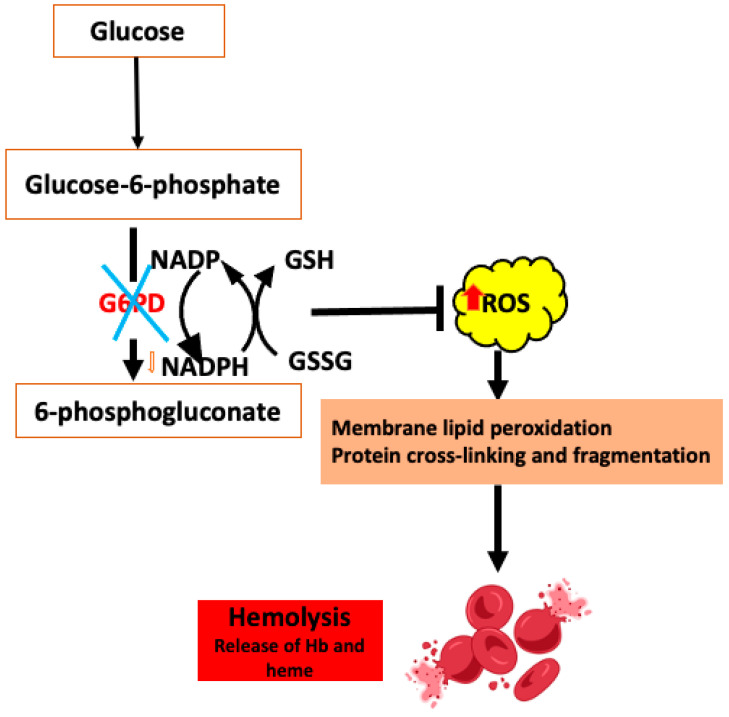
The impact of glucose 6-phosphate dehydrogenase deficiency on red blood cells. ROS: reactive oxygen species, GSH: glutathione, GSSG: glutathione disulfide.

The term “oxidative stress” was first introduced by Helmut Sies to describe an imbalance between oxidants and antioxidants, with oxidants dominating and eventually leading to molecular damage [54]. The dual nature of reactive species is well known; they can either be harmful or beneficial to living organisms. ROS/RNS are frequently produced during normal physiological reactions including immune defense and aging, and in various pathological conditions, including inflammation. Environmental factors, such as infections, drugs, and industrial toxicants, may also contribute to increased ROS production. Additionally, inadequate performance of antioxidant systems can cause OS, which is linked to damage and oxidative modification of essential molecules, including proteins and lipids, ultimately damaging RBCs themselves or other vasculature components and, thus, impairing their functions (Figure 2) [55]. These consequences include the degradation of Hb and other proteins, the disturbance of ionic homeostasis (Ca^2+^), the formation of neoantigens, hindered RBC deformation, interference with erythropoiesis, and the enhanced exposure of phosphatidylserine. RBCs have a resilient systemic redox buffering system that helps them resist oxidation. However, any failure in the antioxidant defense or conditions that escalate oxidant production can have severe effects on RBCs at a subcellular level. RBC membranes contain high levels of polyunsaturated fatty acids, which make them particularly vulnerable to peroxidation, causing a loss of membrane integrity and decreased activity of membrane enzymes like ATPase and acetylcholinesterase [56,57].

OS is a common patho-physiological mechanism that can trigger several cell signaling pathways. This condition is associated with the development of various chronic diseases. These include neurodegenerative, age-related, and cardiovascular diseases, as well as chronic kidney disease [55,56,57].

### 5.1. Oxidative Stress and Red Blood Cell Diseases

Red cell diseases are a group of inherited or acquired erythrocyte disorders that affect the synthesis, function, or makeup of RBCs. These problems may lead to a variety of clinical symptoms, including hemolysis, anemia, inflammation, and reduced oxygen-carrying capacity. OS significantly impacts RBC diseases and the aging process. The level of OS in RBCs is determined by the balance between pathophysiological mechanisms that generate ROS and the antioxidant systems, both enzymatic and non-enzymatic, present in RBCs. Due to their high oxygen-carrying capacity and lack of organelles, RBCs are particularly vulnerable to oxidative damage. This OS can hinder the deformability of RBCs, resulting in reduced oxygen delivery and the premature removal of cells from circulation. Various biomarkers, including heme degradation products, can reflect the levels of OS in RBCs.

RBC storage for transfusion induces biochemical and biological modifications, collectively known as the “storage lesion” [58]. These lesions include alterations in energy metabolism and redox balance, resulting in OS and subsequent protein and lipid modifications [59]. Hb oxidation at functional amino acid residues is a key aspect of these storage-induced changes. Morphological alterations, such as membrane blebbing and vesiculation, also occur during storage [58,59]. These lesions may affect RBC functionality and post-transfusion viability, potentially compromising transfusion efficacy. Understanding these processes is crucial for improving blood storage methods and transfusion outcomes. Hypoxic storage has been shown to mitigate these lesions in both human and murine RBCs, improving energy metabolism and post-transfusion recovery [60]. Recent studies have investigated the effects of antioxidant supplementation on stored RBCs for transfusion. The addition of ascorbate and N-acetyl cysteine to stored RBCs improved overall quality, including increased glutathione and α-tocopherol levels and reduced lipid oxidation [61]. However, high-dose intravenous vitamin C infusion led to increased methemoglobin formation and peroxiredoxin 2 oxidation in RBCs, suggesting potential detrimental effects at high concentrations [62]. Combining vitamin C with vitamin E or L-carnitine in storage solutions showed synergistic benefits, including improved Hb levels, enhanced antioxidant enzyme activity, and reduced lipid peroxidation [63,64]. These studies highlight the potential of antioxidant supplementation in improving RBC storage quality, but also emphasize the need for the careful consideration of dosage and combination strategies to optimize benefits and minimize potential adverse effects.

Furthermore, OS plays a role in the pathophysiology of several RBC disorders, such as sickle cell disease (SCD) [65] and glucose-6-phosphate dehydrogenase deficiency (G6PD) [66]. This section looks at several RBC diseases where this equilibrium is greatly impacted by either reduced antioxidant capacity or increased ROS production. Understanding the mechanisms behind OS in RBCs is crucial for the development of targeted therapies and for assessing overall health.

#### 5.1.1. Deficiency in Glucose 6-Phosphate Dehydrogenase

G6PDH catalyzes the first step in the pentose–phosphate pathway, oxidizing glucose-6-phosphate to 6-phosphogluconate and reducing NADP to NADPH, to provide reducing equivalents to different antioxidant systems. G6PDH deficiency (G6PD) is a highly polymorphic chromosomal X-linked hereditary disease characterized by reduced enzyme activity. Higher susceptibility to OS caused by drugs, anesthetics, infections, and metabolic diseases and decreased NADPH levels in RBCs from G6PD patients can lead to hemolytic anemia (Figure 2) and other health problems (reviewed in [66]).

Interestingly, a number of drugs and chemicals that induce hemolytic anemia in G6PD patients are unable to induce RBC hemolysis in vitro [67], supporting the notion that other genetic factors contribute to the hemolytic phenotype [68]. Recent studies have investigated the impact of G6PD deficiency on RBC storage and transfusion outcomes. A study by Dinarelli et al. found that RBCs from G6PD patients stored for 6–12 days had an unexpectedly decreased susceptibility to hemolysis. The authors claim that the metabolic control exhibited by these aged RBCs led to a reduction in energy consumption and an increase in stress tolerance [69]. However, further patient-centered studies are required to validate this hypothesis. These results also contradict those of Francis et al., who discovered that, after 42 days of storage, the post-transfusion RBC quality of G6PD patients is significantly lower than that of control subjects [70]. This aligns with Karafin and Francis, who reported decreased quality and increased hemolysis in G6PD-deficient RBCs post transfusion. Hemolytic anemia in G6PD patients can be caused by bacterial [71] and viral [72] infections, most likely by triggering the production of ROS by circulating phagocytes.

#### 5.1.2. Deficiency in Pyruvate Kinase

**Figure 3:**
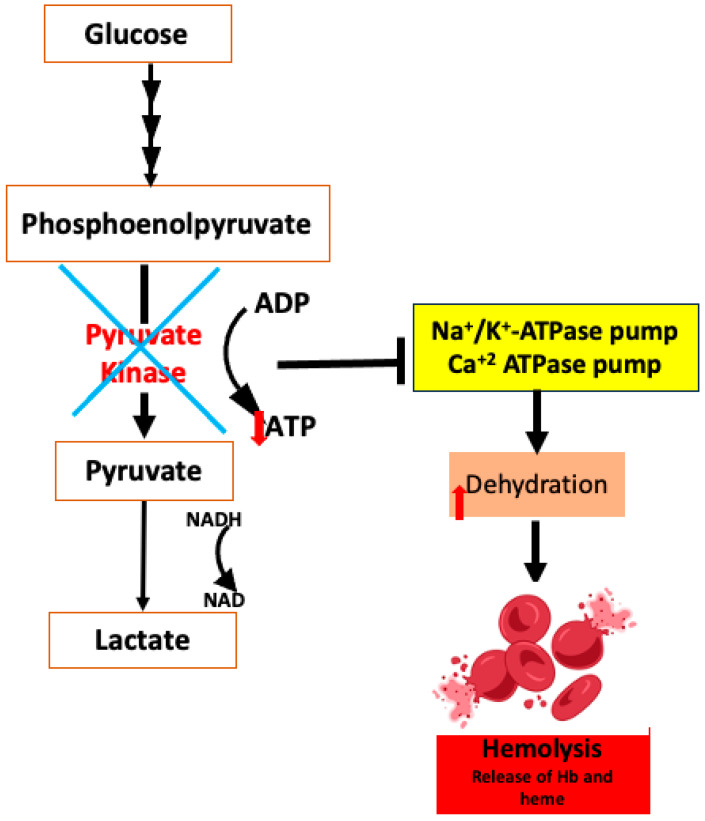
The impact of pyruvate kinase deficiency on red blood cells.

Pyruvate kinase (PK), a crucial enzyme in the glycolytic pathway, catalyzes the conversion of phosphoenolpyruvate (PEP) into pyruvate and generates ATP. Pyruvate kinase deficiency (PKD), an autosomal (chromosome 1q21) recessive genetic disorder that affects the ability of RBCs to make energy, is the cause of hemolytic anemia in different degrees (Figure 3) [73]. Since mature RBCs lack mitochondria, they can only generate ATP through glycolysis. As a result of impaired or reduced PK activity, ATP levels—which are crucial for maintaining the integrity and deformability of the cell—dramatically decline in PKD patients [74]. The main RBC membrane pumps that control the movement of calcium, sodium, and potassium across the RBC membrane are P-type ATPase pumps, whose activity is reliant on the amount of ATP present. The ion balance shifts when these pumps are not working as much, which results in a water leak and the dehydration of the RBCs [75]. The changed membrane properties of PKD patients’ RBCs lead to hemolytic anemia and increase their susceptibility to early splenic destruction [76]. Along with the elimination of altered mature RBCs, patients with PKD also have fewer reticulocytes, which are especially susceptible to low ATP levels. Another consequence of PK deficiency is the accumulation of glycolytic intermediates, such as 2,3-biphosphoglycerate (2,3-BPG). This may help to partially compensate for anemia by enhancing tissue oxygenation and decreasing the affinity of O_2_-hemoglobin [77].

A metabolomic approach was developed by Roy et al. [78] to characterize specific changes in the metabolic pathways of PKD patients. According to the study, OS markers such as sulfur-containing compounds, polyamines, and deaminated purines are more prevalent in RBCs from PKD patients and are associated with a greater number of pentose phosphate pathway metabolites. These individuals also showed higher levels of poly- and highly unsaturated fatty acids, as well as acyl carnitine [78].

#### 5.1.3. Sickle Cell Disease

An aberrant hemoglobin S (HbS) that undergoes deoxygenation-dependent polymerization is produced in SCD, an inherited genetic condition [65]. RBCs undergo shape distortion, cell stiffness, cell membrane change, and fragility as a result of repeated cycles of HbS polymerization, which ultimately lead to intravascular and extravascular hemolysis. SCD pathophysiology seems to be more complicated and involves a broad network of molecular and cellular partners behind this main pathogenic mechanism that has been characterized. Indeed, in addition to HbS polymerization, an imbalance in redox status is also seen in SCD, which is caused by a rise in ROS and/or RNS generation coupled with a malfunction in the antioxidant systems. Peroxynitrite, for instance, contributes to the oxidation and nitration of a number of intracellular targets, including lipids, proteins, and thiols, which can result in DNA damage, the disruption of cell signaling, and cell death (reviewed in [79]). Sickle RBCs, activated neutrophils, platelets, and endothelial cells (ECs) can all contribute to OS in SCD. This pro-oxidant environment has been explained by a number of erythroid and non-erythroid mechanisms, including (1) HbS auto-oxidation, (2) heme and iron release, (3) increased activity of endothelial xanthine oxidase (XO) and NADPH oxidase, (4) decreased NO· bioavailability, and (5) erythroid mitochondrial retention [80].

##### HbS Autoxidation

Due to its extreme instability, HbS is susceptible to autoxidation when exposed to oxygen. The reaction yields MetHb, which no longer binds oxygen, and O_2_^·−^, which dismutates to H_2_O_2_ [5]. By resulting in hemolysis, oxidative damage to the RBC membrane, lipid and protein oxidation, and the release of toxic heme, this intensifies the pro-oxidative milieu. The most prevalent protein in the RBC membrane is phosphorylated; various erythroid proteins are ubiquitinated, Cys93 of the β globin is oxidized, and Lys96 and Lys145 are ubiquitinated, among other post-translational changes that occur in Hb under HbS-induced OS. ROS changes band 3, which clusters and separates from membrane/cytoskeleton complexes, resulting in RBC membrane disruption and, potentially, microparticle production [81]. Numerous investigations have demonstrated the part microparticles play in a number of SCD consequences, including renal failure and vaso-occlusion [82].

##### Hemolysis: Heme and Iron Release

Frequent sickling/unsickling cycles result in hemolysis, which increases the vascular oxidative burden and releases extracellular hemoglobin, free heme, and free iron, all of which are highly detrimental to the vasculature [83]. Actually, OS generated at the erythroid level can affect not just RBCs, but also neutrophils, monocytes, and endothelial cells. Through a variety of physiological pathways involving many receptors, such as P2X7, toll-like receptor 4 (TLR4), and other unidentified receptors, hemolyzed red blood cells release heme and ATP, which act as damage-associated molecular patterns (DAMPs) and activate endothelial cells, neutrophils, and macrophages. These activation mechanisms also result in the creation of pro-inflammatory mediators and adhesion molecules at the cell surface, which worsen the pro-inflammatory and oxidant environment. This could eventually lead to vaso-occlusion and other issues related to SCD [84].

##### NADPH Oxidase and XO Activity

One of the main enzymes in leukocytes, RBCs, and endothelial cells that produces O_2_^·−^ is NADPH oxidase. Erythroid NADPH-produced ROS exacerbate erythroid failure by stiffening cells, which heightens hemolysis [85]. A significant portion of the synthesis of O_2_^·−^ and H_2_O_2_ is also attributed to xanthine oxidase (XO). Although its origin is yet unknown, SCD plasma exhibits increased levels of its activity [86].

##### NO· Bioavailability

Vascular physiology and homeostasis are significantly impacted by NO·. Notably, it acts as a vasodilator on smooth muscle cells and suppresses the development of selectin family members on endothelial cells, including ICAM-1 and VCAM-1 [87]. Moreover, NO· may prevent the activation of platelets [88]. One intriguing theory is that the soluble guanylate cyclase (sGC) might play a role in regulating the RBC’s deformability [89]. In SCD, NO· is scavenged during hemolysis, which lowers the blood bioavailability of free extracellular hemoglobin. Furthermore, by combining to form ONOO^−^, the synthesis and release of O_2_^·−^ may aid in the reduction in NO· [90]. Consequently, the regulation of vascular tone and the production of adhesion proteins are adversely affected by the reduced NO· bioavailability in SCD.

##### Erythroid Mitochondrial Retention

RBCs from SCD patients have been shown to preserve mitochondria by Jagadeeswaran et al. [91], and other groups have verified this [92]. The mechanism of action of these mitochondria is still up for debate, though. According to certain research, they were still operational, and higher ROS levels were linked to mitochondrial retention. However, some of these findings were found in a population of erythroid circulating cells, which may also include reticulocytes or immature RBCs, or in a mouse model of sickle cell disease [92]. These conserved mitochondria were not shown to be active in adult RBCs in another investigation [93]. The mechanism underlying this mitochondrial retention and the direct connection between it and the elevated OS in SCD remain unclear.

## 6. Conclusions and Future Direction

RBC effectiveness can be impacted by a number of factors. These include a limited ability to repair damaged elements due to the loss of protein expression during erythropoietic maturation, high levels of molecular O_2_ bound to Hb, which results in an abundance of oxidants, and high levels of iron within the prosthetic group of Hb, which can cause ROS production via the Fenton reaction. To fight these challenges, RBCs have enzymatic and non-enzymatic antioxidant systems that protect their structural proteins, metabolic enzymes, channels, and membrane lipids from oxidative damage. These mechanisms are essential for keeping Hb in its reduced oxygen-binding form, which is required for cellular survival, and they also reduce extracellular antioxidants through a transmembrane electron transport pathway. Antioxidant system dysfunction can lead to hemolytic anemia. The antioxidant mechanisms of RBCs also contribute to the overall systemic homeostasis of redox equilibrium.

To mitigate OS and its related diseases, specific therapeutics must be developed, which requires ongoing study into the redox biology of RBCs. Future research could examine pharmacological and genetic therapies to increase RBCs’ antioxidant capability. Promising treatment options may include looking into the function of new antioxidants and the possibility of using gene therapy to address enzyme pathway deficits. Furthermore, studies on how nutrition and activity affect RBC redox balance may shed light on preventive measures for groups that are already at risk. In order to translate these discoveries into clinical practice and eventually improve the quality of life for patients with OS-related disorders, cooperation between researchers, doctors, and industry partners will be essential.
